# Liposome–Hydrogel Composites for Controlled Drug Delivery Applications

**DOI:** 10.3390/gels10040284

**Published:** 2024-04-22

**Authors:** Roya Binaymotlagh, Farid Hajareh Haghighi, Laura Chronopoulou, Cleofe Palocci

**Affiliations:** 1 Department of Chemistry, Sapienza University of Rome, Piazzale Aldo Moro 5, 00185 Rome, Italy; 2 Research Center for Applied Sciences to the Safeguard of Environment and Cultural Heritage (CIABC), Sapienza University of Rome, Piazzale Aldo Moro 5, 00185 Rome, Italy

**Keywords:** hydrogels, liposomes, nanoparticles, drug delivery, encapsulation, cancer

## Abstract

Various controlled delivery systems (CDSs) have been developed to overcome the shortcomings of traditional drug formulations (tablets, capsules, syrups, ointments, etc.). Among innovative CDSs, hydrogels and liposomes have shown great promise for clinical applications thanks to their cost-effectiveness, well-known chemistry and synthetic feasibility, biodegradability, biocompatibility and responsiveness to external stimuli. To date, several liposomal- and hydrogel-based products have been approved to treat cancer, as well as fungal and viral infections, hence the integration of liposomes into hydrogels has attracted increasing attention because of the benefit from both of them into a single platform, resulting in a multifunctional drug formulation, which is essential to develop efficient CDSs. This short review aims to present an updated report on the advancements of liposome–hydrogel systems for drug delivery purposes.

## 1. Introduction

### 1.1. Liposomes

**Figure 1:**
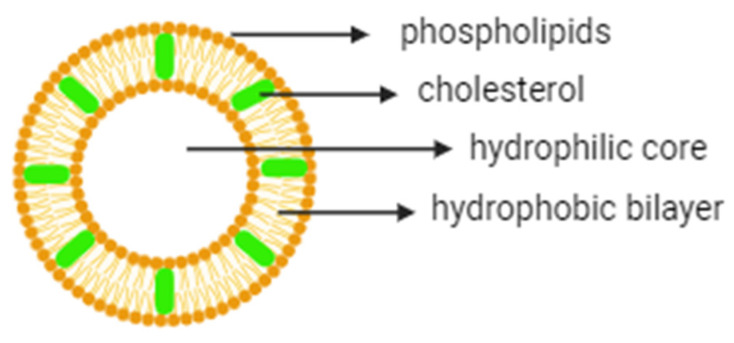
The general structure of a liposome.

Conventional drug delivery systems have inherent limitations such as poor targeting and low therapeutic indices, which result in systematic side effects and increase costs and duration of the therapy. To overcome these drawbacks, various nano-delivery systems have been developed for different therapeutic applications. Among them, liposomes (self-assembled lipid vesicles) are, to date, one of the most studied nanosystems for clinical applications [1,2]. Liposomes are self-assembled phospholipid bilayer (unilamellar) or multiple-bilayer (multilamellar) structures that form an internal hydrophilic center with overall diameters ranging from 30 nm to the micrometer scale (Figure 1) [3]. The liposomology field was introduced in the mid-1960s in Cambridge [4] when Alec Bangham’s group first described the liposome structure [5]. Since then, liposomes have been extensively investigated as delivery vehicles for a variety of molecules such as proteins, drugs, nucleic acids and imaging agents. As outstanding drug vehicles, liposomes protect encapsulated molecules against physiological degradation, prolonging the half-life of the drug; they can also provide excellent biocompatibility, safety and controlled drug release kinetics [6,7]. More importantly, liposomal delivery systems can be designed for passive and/or active targeting of diseased sites to elevate the tolerated dose and decrease the adverse side effects connected with the use of free drugs [8,9].

As mentioned above, liposomes are biodegradable, biocompatible, non-toxic and they are composed of amphiphilic non-immunogenic compounds (such as cholesterol and phospholipids). They are able to improve solubility and tissue penetration of both lipophilic and hydrophilic drugs. These features have allowed their successful exploitation in numerous areas of nanomedicine, and, at present, twenty liposome-based formulations have been approved by the FDA (US Food and Drug Administration) and/or EMA (European Medicines Agency) (Table 1) [10]. It is worth mentioning that this list excludes nationally authorized products in Europe, generics and lipid complexes (e.g., Onpattro, Amphotec and Abelcet). Among them, Doxil (Doxorubicin HCl–liposome injection) was the first FDA-approved liposomal-based formulation in 1995; 57% of these products were approved before 2010. Generally, the main area of application of liposome-based drugs is cancer therapy; however, infection treatment, anesthesia, photodynamic therapy and vaccination are witnessing an increasing use of liposomal formulations. Such preparations are mainly used as lyophilized powders or sterile suspensions, and they can be administered by different routes, including intramuscular, intravenous infusion, intrathecal injection, oral inhalation, epidural and local infiltration [11,12,13]. It should be also mentioned that liposomes are commonly used in different cosmetic products, as described in some recent reviews [14,15].

Transferosomes are ultra-deformable carriers consisting of a phospholipid bilayer with an edge activator (e.g., sodium deoxycholate, Tween^®^ 80, Span^®^ 80) and an ethanol/aqueous core [16]. Based on the lipophilicity of the cargo, it can be encapsulated in the lipid bilayer or within the core. Transferosomes have shown to have an important advantage over liposomes due to their ability to reach intact deeper regions of the skin after local administration (via paracellular and intercellular mechanisms across the corneocytes); consequently, they can deliver higher drug concentrations in transdermal applications. Phosphatidylcholine (C18, the most abundant lipid component of cell membranes) is the main component of most transferosomes and provides high tolerance for the skin, lowering undesirable effects (e.g., hypersensitive reactions). Both small and large drugs have been successfully encapsulated within transferosomes, such as phytocompounds like apigenin or sinomenine against leukemia and rheumatoid arthritis, respectively. Also, macromolecules like insulin have been entrapped in transferosomes. The key parameters to manufacture optimal transferosomal formulations (with nanometric sizes and high drug loading) are the optimal ratio between their components and manufacturing parameters (e.g., cost, reproducibility, high mechanical stability, etc.). Applying quality by design (QbD), specifically design of experiments (DoE), is necessary for understanding the interplay among all these parameters for lab-scale preparation as well as for its scale-up. 

Clinical studies have confirmed the tolerability of transferosomal formulations; however, more studies are still necessary to develop standard protocols in combination with other technologies to enhance the permeation, such as electroporation, iontophoresis and micro-needles, to facilitate drug delivery across the skin [17,18,19,20,21]. In general, there are many strategies yet to be explored in order to modify the properties of transferosomes and close the gap between lab-scale knowledge and clinical technology, for example, in order to address the long-term stability challenges of transferosomes in liquid media. Regarding the clinical trials of a licensed topical ketoprofen transferosomal gel (Diractin^®^, licensed by the Swiss Regulatory Agency in 2007), promising results have been obtained in the alleviation of symptoms in osteoarthritis with non-severe skin and subcutaneous tissue side effects [22]. However, six months after its approval, the product was withdrawn from the market, probably due to the higher cost of the medicine (compared with conventional gels) linked to its expensive production process. This highlights the need for precise formulation design for the development of sustainable industrial manufacturing.

Liposome preparation methods can be divided into two main categories of (1) conventional and (2) innovative methods, as summarized in Table 2. Lamellarity, morphology, composition and size strongly depend on the preparation method. There are standardized and optimized conventional methods, especially for laboratory-scale preparation. Conventional methods include the following:

(1) Thin-lipid film hydration is the most common methodology, used to prepare different structures, including small unilamellar (SUVs), multilamellar (MLVs) or giant unilamellar (GUVs) vesicles [23,24,25]. The limitations of this method are broad size distribution, high temperature, possible liposome degradation upon sonication or low drug encapsulation yield.
(2) Reverse-phase evaporation [26] is the second most used technique to obtain large unilamellar vesicles using water-in-oil formation from a surfactant/lipid mixture with an aqueous solution of the drug. The organic solvent is then removed under reduced pressure; however, the trace amounts of organic solvent in the final formulation can influence vesicle stability.
(3) Solvent injection is based on the injection of an organic phospholipid solution into an aqueous phase of the selected drug at a temperature above the organic solvent boiling point. Vesicle size can be controlled with this method; however the presence of organic solvent in the final product is considered a major disadvantage for this approach.

To address the limitations of such traditional methods, more efficient novel approaches are being developed. In this regard, microfluidic technology has evolved at both lab and industrial scales to obtain monodisperse liposomes [27,28] by controlling parameters such as micro-channel size and flow rates. The main advantages of this method are high yields, efficient liposomal distribution and high drug encapsulation efficiencies. However, for scaling-up, the device fabrication and optimization of different fluid phases and multiple fluid inputs may be challenging and are, therefore, considered the main limitations of microfluidic technologies.

Microfluidic devices are microscale circuits used to synthesize nanoparticles as well as liposomes [29]. By finely controlling the mixing of phases, microfluidic devices provide the possibility to optimize the quality and encapsulation efficiency of liposome-based drug delivery systems [30]. Compared to conventional technologies (e.g., solvent evaporation), the reproducibility of the synthesis is generally improved thanks to the automation of this technique. The continuous nature of this method can save the cost and time of studying scarce or costly materials, allowing optimization even at low volumes [31]. It should be mentioned that microfluidic devices usually have complex engineering, which limits their scale-up. This barrier may be addressed by using 3D printing technology, which is a more cost-effective way to improve the channel resolution with a variety of commercially available materials possessing suitable properties, such as being biocompatible, transparent and non-fluorescent [32]. In this regard, the use of PDMS chips for the manufacturing of nanomedicines has been extensively reviewed [33].

Using high-resolution 3D printing based on stereolithography or fused deposition modeling, reliable patterning of channel features with ~200 µm dimensions has been performed, providing the possibility to prepare high-quality nanomedicines (<100 nm at a production rate of 4 mg/min) [34]. This may be achieved thanks to the development of flow-focusing micro-channels that support large volumetric flow rates and high-throughput nanoparticle synthesis [35] with tunable dimensions [23]. However, solvent removal and the presence of free drugs remain a challenge for continuous manufacturing using this method. The applicability of 3D-printed microfluidic chips for the manufacturing of nano-delivery systems has recently been demonstrated, and a few examples are presented [36,37,38]. For example, curcumin-loaded liposomes were synthesized using FDM-printed chips (with 1000 µm channels), in which liposomes of about 200 nm and 99% encapsulation efficiency were obtained [39].

**Table 2:** Main liposome preparation methods [1].

Preparation Method	Particle Size (nm)	Advantages	Disadvantages	Ref.
Thin-lipid film hydration	100–1000	Most widely used method	Low encapsulation efficiencies, sonication, temperature exposure, heterogeneous size distribution	[40]
Reverse-phase evaporation	100–1000	High encapsulation efficiency	Organic solvent traces	[41]
Solvent injection (ether or ethanol)	70–200	Ability to control vesicle size	Dilution of liposomes, heterogeneous populations, use of high temperatures	[42]
Microfluidic technologies	100–300	Synthesis of monodisperse liposomes, high encapsulation efficiency	Large-scale fabrication may be complex and requires optimization	[43]
Supercritical reverse-phase evaporation	100–1200	Environmentally friendly process, high encapsulation efficiency	High pressures and temperatures	[44]
Spray drying	100–1000	Control over particle formation, easily translated to large-scale production	Expensive and time-consuming	[45]
Membrane contactor technology	~100	Homogeneous and small sizes, high encapsulation efficiency, simplicity for scaling-up	Hydrophilic drug encapsulation needs optimization	[46]
Crossflow injection	~50	Liposomes of defined size	Vessicle instability due to residual solvent	[47]

Other effective industrial techniques have been developed, like supercritical reverse-phase evaporation [48], which uses supercritical CO_2_ as a non-toxic and non-flammable fluid to dissolve phospholipids, providing an environmentally friendly process and an excellent alternative to the use of organic solvent methods for preparing liposomes. Considering this, this technology usually has higher encapsulation efficiencies than conventional procedures. Other alternative methods, including crossflow injection [49], membrane contactor technology [50] and spray drying [51], have also been described for liposome industrial production, thanks to their cost-effectiveness and short duration processes.

The main advantages and disadvantages of conventional liposome applications are summarized in Table 3. Industrial applications of liposomes are generally focused on the preparation of drug delivery systems and vaccine adjuvants in medicine, as well as support matrices for various ingredients and penetration enhancers in cosmetics. Furthermore, liposomes are used as signal enhancers/carriers in medical diagnostics and analytical biochemistry, as solubilizers for various ingredients, etc. [52].

### 1.2. Hydrogels

Hydrogels are 3D cross-linked polymer networks with high water-absorbing abilities similarly to body tissues, which allows them to encapsulate drugs and protect them in physiological conditions [53,54,55,56,57,58,59,60,61,62]. Hydrogels can be classified based on their characteristics and structure, for instance (1) their charge (cationic, neutral, anionic or ampholytic hydrogels); (2) the nature of their side groups (e.g., charged or neutral); (3) their physical structural parameters (e.g., hydrogen-bonded structures, amorphous or semi-crystalline, supramolecular structures, hydrocolloidal aggregates); (4) the nature of cross-links (e.g., physical or chemical); (5) the preparation method (e.g., homo- or co-polymers); and (6) their origin (e.g., synthetic or natural) [63]. Regarding the physical forms of hydrogels for therapeutic applications, they can be prepared as pressed powder matrices (e.g., pills and capsules), microparticles (e.g., wound treatment), solid molded forms (e.g., contact lenses), beads (e.g., drug delivery), coatings (e.g., implants or catheters) and membranes or sheets (e.g., a reservoir in a transdermal delivery patch). Since the introduction of poly-2-hydroxymethacrylate (PHEMA)-based hydrogels for contact lens applications in 1960 [64], an increasing number of researchers have worked to develop not only polymeric but also natural-based hydrogels to be used for therapeutic applications such as controlled drug delivery systems. The delivery mechanism of hydrogels is usually controlled by passive diffusion, which strongly depends on hydrogel structure (e.g., hydrogel pore size, cross-linking degree, stimuli-sensitive hydrogel capacity, etc.). However, for clinical applications, there is a possibility of undesired and immediate drug release upon contact with the medium, which may increase the local concentration of the drug (“dose dumping”), causing an unexpected in vivo toxicity. To minimize this effect, several strategies have been reported, which are based on structural modifications of the hydrogel and/or the drug [65,66,67].

### 1.3. Integration of Hydrogels and Liposomes (Liposomes–Hydrogels)

Despite the development of both liposome- and hydrogel-based technologies, sometimes their drug delivery applications are limited by several shortcomings such as instability and rapid degradation. To address these issues and improve their efficacy, the integration of hydrogels and liposomes (liposomes–hydrogels) could represent a promising strategy to minimize fast drug release, especially in special fields including sustained drug delivery and wound therapy. More importantly, both hydrogels and liposomes may improve each other structurally, for instance, a hydrogel can modify the mechanical stability and membrane integrity of encapsulated liposomes. These things considered, the interaction of liposomes with hydrogels, lipid composition and liposome concentration may improve the swelling/deswelling properties of hydrogels and their rheology [68,69], consequently modulating the drug release profiles from the whole hybrid system. So, the combination of liposomes and hydrogels could improve both drug formulation and drug administration routes.

Regarding the preparation methods for liposomes–hydrogels, they are mainly based on incubating pre-formed hydrogels with liposomes. However, the self-assembling of phospholipids and bilayer stabilities of liposomes should be considered [70]. This combination strategy has been successfully employed in the preparation of a wide number of liposomes–hydrogels (both synthetic and natural hydrogels) to obtain stimuli-responsive hybrid materials.

For clinical applications, the use of biodegradable and biocompatible hydrogels has attracted a paramount importance over the last few decades. The first liposome–hydrogel system of this kind was introduced by Weiner’s group in 1985 [71], containing two peptide hormones (growth hormone and insulin) into a collagen hydrogel. The authors reported slow release rates of the hormones and observed an improved release from liposome–hydrogel formulations, compared to liposomes. Since then, hybrid liposome–hydrogel systems have emerged as a promising approach for obtaining advanced drug delivery systems. This review describes the most relevant examples of liposomes encapsulated in different types of hydrogels, including peptide-based, biopolymeric and synthetic polymer hydrogels, for drug delivery applications.

## 2. Liposomes Encapsulated in Different Types of Hydrogels

### 2.1. Liposomes Encapsulated in Peptide/Amyloid Hydrogels

In clinical application, there are many biological barriers that limit the successful delivery of a drug to its target site. This limitation can be improved designing advanced drug delivery systems, which modify the targeting ability, solubility, metabolism and cytotoxicity of the drug [72]. Doxorubicin (Dox) is a commonly used anticancer drug, which intercalates within DNA to inhibit topoisomerase II [73]. Despite its therapeutic applications, the clinical use of Dox is restricted by its dose-limiting toxicity, resulting in cardiotoxic and myelosuppression side effects that increase cardiovascular risk [74]. Furthermore, a dose-dependent cardiotoxicity of Dox appears from the very first administration and increases for each following anthracycline cycle. To overcome these drawbacks, different nanoformulations encapsulating Dox have been proposed as an alternative strategy for its administration. Currently, two Dox liposomal formulations, Caelyx^®^/Doxil^®^ and Myocet^®^ and their bioequivalent formulations are used in clinical settings. The liposomal spatial confinement of Dox allows altering the biodistribution of the drug, minimizing its toxicity, increasing its half-life and therapeutic index, while improving its pharmaceutical profile, thus leading to increased patient compliance. The integration of liposomes into amyloid hydrogels has been explored for the sustained delivery of Dox [75]. Amyloid hydrogels are made of insoluble fibrils, containing highly ordered protein self-assemblies, which provide high mechanical and chemical stability. Such systems have been proposed as promising drug nanodepots [76]. In this regard, Trusova et al. designed a new liposome–amyloid hydrogel for delivering Dox and a hydrophobic europium coordination complex [77]. Two types of liposomes were developed, containing (1) the lipid phosphatidylcholine (PC) and (2) its mixture with the anionic lipid cardiolipin (CL, 10 mol %), as well as two types of amyloid hydrogels, composed of (1) bovine serum albumin (BSAF) and (2) egg yolk lysozyme (LzF). The results revealed that the negative charge of albumin fibrils of the BSAF hydrogels facilitates Dox encapsulation into PC multilamellar liposomes but shows the opposite effect on CL-type liposomes. Conversely, LzF hydrogels showed no sensitivity to the presence of fibrillar proteins in CL10/Dox/LzF and PC/Dox/LzF systems. Regarding the hydrophobic europium complex, neither BSAF nor LzF hydrogels affected its encapsulation into liposomes. Therefore, this study highlighted the increasing Dox payload efficiency by using liposome–amyloid hydrogels.

**Figure 2:**
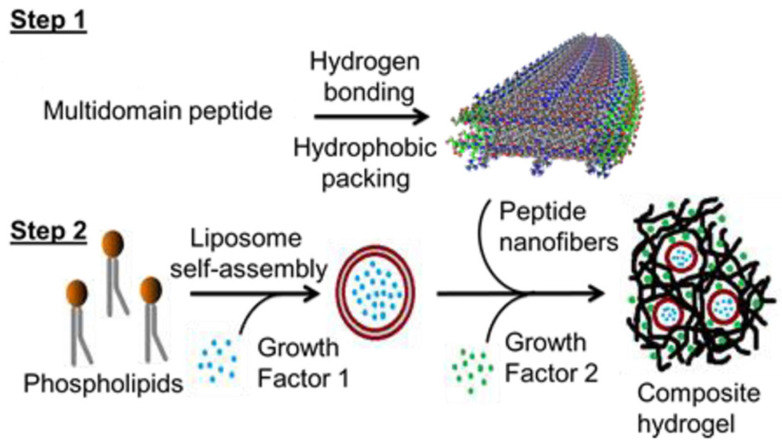
Orthogonal self-assembly combining liposomes, growth factors and MDP fibers. Reprinted from ref. [78]; Copyright © 2014 American Chemical Society, Open Access.

In 2014, Wickremasinghe et al. synthesized a unique hydrogel by the stepwise self-assembly of liposomes (made of dipalmitoylphosphatidylcholine (DPPC), dipalmitoylphosphatidylglycerol (DPPG) and cholesterol) and multi-domain peptide (MDP) fibers [78] to provide the controlled release of desired cytokines and growth factors. The self-assembled peptide, K(SL)3RG(SL)3KGRGDS, was conjugated with a liposomal system encapsulating three different GFs/cytokines labeled with a reporter molecule (Figure 2) for the controlled release of bioactive factors. The rheological data showed that the hydrogel is not affected by the entrapped liposomes, and the release studies of different growth factors showed a sustained release by liposomes in the hydrogel compared to a rapid release from the pristine hydrogel. This liposome–peptide hydrogel formulation can be further studied in systems where timed cascades of biological signals may be valuable, such as in tissue regeneration applications.

### 2.2. Liposomes Encapsulated in Biopolymeric Hydrogels

Different natural polymers have been used for preparing hydrogels for the inclusion of drug-loaded liposomes, including alginate, collagen, gelatin, chitosan (CS), dextran and fibrin [79,80]. The release profiles of loaded drugs can be easily tuned by modifying liposome–peptide hydrogel parameters (e.g., hydrogel and liposome composition, cross-linker) [81,82,83]. These liposome–hydrogel composite materials can control the release of incorporated low molecular weight drugs [84]. To this aim, Ciobanu et al. synthesized CS/gelatin hydrogels through double cross-linking with sodium sulphate/sodium tripolyphosphate and glutaraldehyde, to entrap MLVs or SUVs of phosphatidylcholine liposomes loaded with calcein (used as a model hydrophilic drug) [85]. This polymeric hydrogel creates a stabilizing network for the liposomal surface to stabilize the liposome–drug system as well as providing prolonged drug release. Various CS/gelatin ratios and different types/amounts of ionic cross-linkers have been studied. The results showed that the release of calcein can be precisely controlled within the range from several days to weeks by tuning the structure of the composite system (i.e., multilamellar or small unilamellar vesicles). Multilamellar liposomes demonstrated a better release behavior, indicating that they remain intact after release from the hydrogel network, due to their enhanced stability provided by the multiple protective layers. However, when small unilamellar liposomes were used, calcein was predominantly released from the hydrogel matrix due to the unilamellar-related instability of the liposomes. Therefore, by tuning hydrogel features (i.e., the type/mount of cross-linking agent and the components ratio) and liposome structures (i.e., lamellarity and size), this study may be extended to regulate drug release kinetics of other water-soluble drugs for various biomedical applications.

In another study, Billard et al. synthesized an innovative liposome–hydrogel composite system made of phosphatidylcholine liposomes (MLVs and SUVs) encapsulated inside a CS hydrogel [86]. This drug delivery system was prepared by suspending liposomes into CS solutions, after which the gelation of the polymer was successfully performed, as confirmed by rheological studies of the composite. The controlled release of this liposome–hydrogel system was studied with carboxyfluorescein (CF, a model water-soluble molecule) encapsulated in liposomes. The results showed that CF release was delayed by the liposome–hydrogel composite (cumulative release of ~70%), compared with the CF hydrogel (cumulative release of ~85%), thanks to the lipid vesicles. The rheological properties of pristine CS hydrogels were not significantly changed by the presence of liposomes. The molecular weight of CS chains and their acetylation degrees can impact hydrophobic/hydrophilic domains of the hydrogel network, thus influencing their potential interactions with liposomes. 

**Figure 3:**
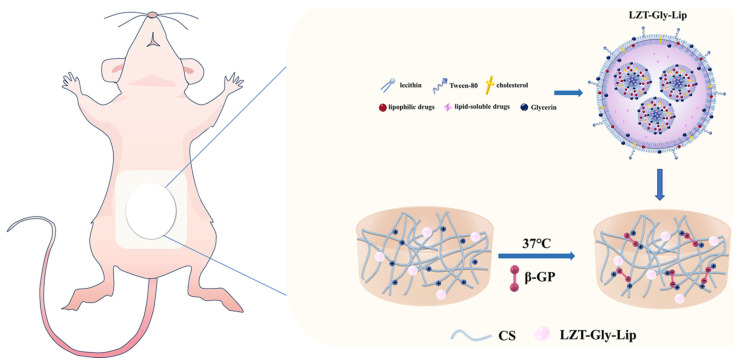
Schematic representation of LZT-Gly-liposome/CS hydrogel system. Reprinted from ref. [87]; Creative Commons Attribution license.

Primary ovarian insufficiency (POI) is recognized by irregular ovulation and reduced estrogen production, often causing lowered fertility or even infertility. To treat POI, the traditional Chinese medicine Liu Zi Tang (LZT) has shown promising results; however, conventional oral administration has some limitations, e.g., the liver’s first-pass effect and gastrointestinal irritation. In 2024, Liu et al. incorporated LZT extracts into a novel glycerol plasmid-liposome/CS hydrogel composite, to prepare a LZT-glycerol plasmid/CS hydrogel (LZT-Gly-Lip/CS Gel) drug carrier to achieve a transdermal controlled-release drug delivery system (Figure 3) [87]. For liposome preparation, egg yolk lecithin, cholesterol and Tween 80 were mixed in anhydrous ethanol as the organic phase. The POI model was tested in rats, comparing LZT-Gly-Lip/CS Gel treatment with the effects of intraperitoneal injection of vinylcyclohexene dioxide (VCD). The rats treated with LZT-Gly-Lip/CS Gel composite showed significant enhancements in serum estradiol concentration, body weight and uterus index, approaching normal levels. These results demonstrated the potential of LZT-Gly-Lip/CS Gel as a transdermal drug delivery system for addressing POI.

The authors introduced this drug delivery system as a promising candidate for various traditional Chinese medicines in the future. However, liposomes–hydrogels face some limitations, such as low drug loading capacity, which should be further studied to identify and implement solutions.

**Figure 4:**
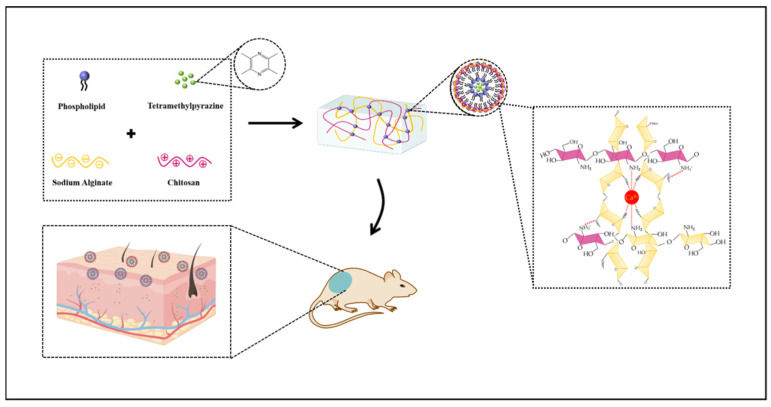
Preparation of tetramethylpyrazine-loaded liposomes modified with sodium alginate and CS hydrogel, applied on the skin of AD-like mice. Reprinted from ref. [90]; Creative Commons Attribution license.

Regarding chronic inflammatory skin diseases, atopic dermatitis (AD, also known as atopic eczema) is characterized by itchy, typically distributed eczema skin injury [88]. Worldwide, around 10% of adults and 20% of children suffer from AD [89]. Among all skin diseases, AD is one of the most challenging encountered by skin care professionals; more importantly, the use of AD drugs often has side effects and patients are vulnerable to bacterial infections, which further complicates treatment. Commonly used drugs (e.g., tetramethylpyrazine (TMP)) show fast metabolism and low bioavailability, which is not effective for the transdermal treatment of AD. In 2024, Xia et al. synthesized a multifunctional liposome–hydrogel delivery system as a promising alternative treatment for AD [90]. For liposome preparation, the authors mixed soybean lecithin and cholesterol in anhydrous ethanol and dissolved them using ultrasounds at 70 °C. They encapsulated TMP into liposomes, followed by surface modification with CS and sodium alginate (ALG) (Figure 4), to prepare TMP–liposome/ALG–CS hydrogels. In vitro experiments showed antibacterial (because of the presence of CS) as well as anti-inflammatory/antioxidant effects (due to the presence of TMP). Furthermore, TMP–liposome/ALG–CS hydrogels provided better skin permeability due to a moist healing environment for AD dry skin, achieving a controlled drug release, which is necessary for treating AD. The 1-Chloro2,4-dinitrobenzene was used to induce the lesions for in vivo experiments in mice. TMP–liposome/ALG–CS hydrogels alleviated oxidative stress and increased SOD activity in treated mice.

**Figure 5:**
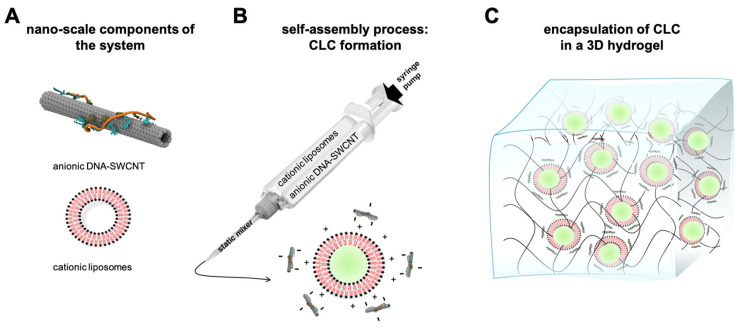
DNA-wrapped single-walled carbon nanotubes and liposomes are self-assembled to form carbon nanotube–liposome complexes (CLCs) by electrostatic forces and then encapsulated in a 3D hydrogel matrix: (**A**) nano-scale components of the system: anionic DNA-wrapped SWCNTs and cationic liposomes; (**B**) DNA-wrapped SWCNTs and liposomes are mixed at different ratios by using a syringe and static mixer and CLCs self-assemble at this step; and (**C**) CLCs are then encapsulated into a covalently cross-linked alginate hydrogel. Reprinted from ref. [91]; Copyright 2021 American Chemical Society.

In another study, Madani et al. synthesized a controllable drug delivery system by combining single-walled carbon nanotubes (SWCNTs) with hydrogels and liposomes [91]. They incorporated carbon nanotube–liposome complexes (CLCs) into a 3D alginate hydrogel for an optically controlled drug delivery system (Figure 5). To prepare the liposomes, the authors used a thin-lipid film hydration method to form DOPC/DOTAP 1:1 liposomes (DOPC: 1,2-dioleoyl-sn-glycero-3-phosphocholine; DOTAP: 2-dioleoyl-3-trimethylammonium propane). Fluorescein isothiocyanate dextran (FITC-Dex) was encapsulated into the modified liposome and then incorporated into the alginate hydrogel. Drug release was triggered by an NIR laser specified to the optical resonance of a particular SWCNT species, in which the amount of released FITC-Dex can be tuned by varying the irradiation time. The potential cytotoxicity of CLC and NIR stimulation was studied using the annexin V/propidium iodide apoptosis assay on RAW 264.7 macrophages, and minimal in vitro toxicity was detected.

In 2020, Palmesse et al. reported the synthesis of an injectable poly(ethylene glycol) liposome hydrogel, containing both matrix metalloproteinase-sensitive peptide cross-links and temperature-sensitive liposomes [92]. The liposomes were prepared by dissolving DPPC and DSPE-PEG-Mal in chloroform with a molar ratio of 95:5 (DPPC: 1,2-dipalmitoyl-sn-glycero-3-phosphocholine and DSPE-PEG-Mal: 1,2-distearoyl-sn-glycero-3-phosphoethanolamine-N-[maleimide(polyethylene glycol)-2000]). A lipid film was formed after solvent evaporation using a rotary evaporator at 40 °C. Rheological studies confirmed the mechanical stability of the hydrogel to achieve a range of physically applicable moduli. This thermo- and enzyme-sensitive liposomal hydrogel was used to encapsulate Dox with a high encapsulation efficiency, and a thermo-sensitive release was observed, with complete release after 48 h. This hydrogel composite did not compromise proliferation and viability of both murine and human fibroblasts, supporting its potential application as a thermo-responsive drug carrier for controlled release.

**Figure 6:**
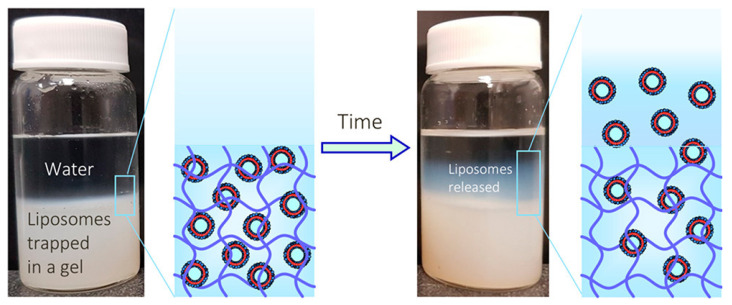
Scheme of the experimental setup and key findings. Initially, (**left**) liposomes are embedded in a hydrogel such as agar, and water is placed above the gel in a vial. Over time (1–3 days), some of the liposomes release out of the gel into the water above (**right**). The release of liposomes can be visually observed as an upward-moving blue front in the water. Reprinted from ref. [93]; Copyright 2020 American Chemical Society.

In 2020, Thompson et al. synthesized 150 nm liposomes from an unsaturated phospholipid (lecithin, soy-phosphatidylcholine or soy-PC) and incorporated them in agar gels (the aqueous phase also contained 0–50% of glycerol, which is an active ingredient in cosmetic products) [93]. When this hydrogel composite was placed in quiescent water, the entrapped liposomes were surprisingly released by the gel into the water (Figure 6), while the hydrogel remained stable. Liposome release rate can be modified by several parameters, e.g., the release kinetics increased with increasing temperature, decreasing concentration of agar and increasing liposome concentration. However, saturated phospholipid liposomes did not release from any gels. A two-step mechanism for liposomal release was proposed: (1) the cross-linking process in the hydrogel is a dynamic process (i.e., breaking and reforming); therefore, large and transient pores (~100 nm) are dynamically formed in the gel matrix and (2) the liposomes are sufficiently deformable and flexible to squeeze through the pores. The saturated phospholipid liposomes did not release out of any gels because of their high rigidity. These liposome–hydrogel formulations could be interesting materials for cosmetics and transdermal delivery, and more detailed modeling should be conducted to study the factors that affect liposomal release rates from such formulations.

**Figure 7:**
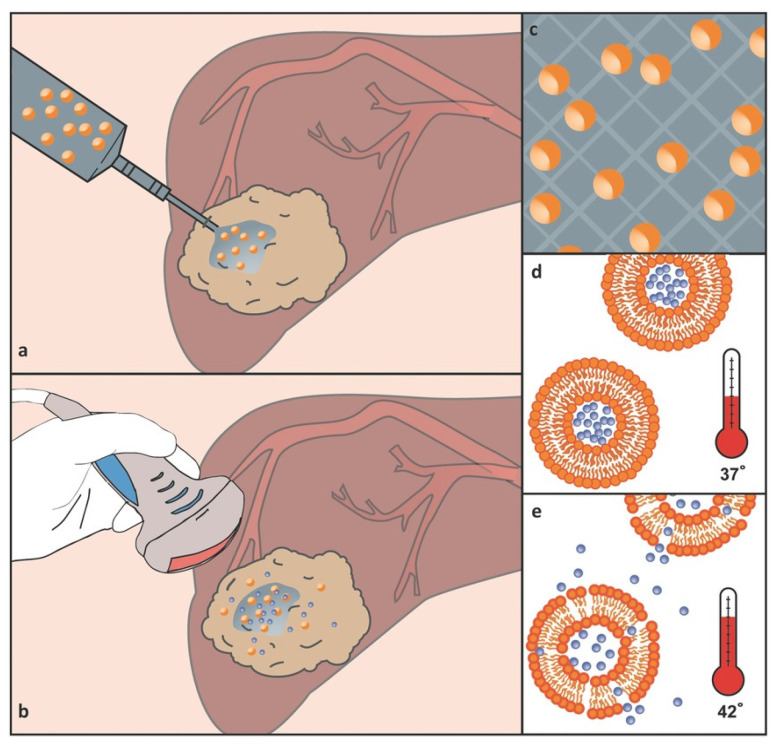
(**a**) Lipogel is fully injectable, consisting of a CS/β-GP thermo-responsive gel hosting a suspension of Dox-loaded thermo-sensitive liposomes. (**b**) In situ release from the gel is controlled using minimally invasive hyperthermia, using high-intensity-focused ultrasounds. (**c**) The majority of liposomes is locked into the gel upon initiation of cross-linking during thermogelation. (**d**,**e**) Liposomes sequester the majority of drug at body temperature, but rapidly become more permeable upon mild hyperthermia and release their drug payload. Reprinted from ref. [106]; Copyright 2014 WILEY-VCH.

One of the main challenges in cancer therapy is the administration of effective concentrations of drugs to a tumor site while minimizing adverse side effects. To this aim, different materials have been developed for achieving both temporal and spatial effective release of the therapeutic molecule at the target site [94,95,96,97,98,99,100,101,102,103]. Among these materials, in situ gelling hydrogel–drug formulations significantly enhance therapeutic effects and overcome the pharmacokinetic limitations of intravenous injection [104,105]. Following this concept, López-Noriega et al. designed a novel thermo-sensitive liposome–hydrogel composite for enabling the localized release of Dox by the incorporation of thermo-sensitive liposomes (loaded with Dox) in a thermo-responsive CS/β-glycerophosphate hydrogel [106]. To prepare the liposomes, dipalmitoylphosphatidylcholine (DPPC), monostearoyl phosphatidylcholine (MSPC) and distearoyl phosphatidylethanolamine-poly(ethylene)glycol 2000 (DSPE-PEG2000) in a molar ratio of 85.3:9.7:5.0 were dissolved in chloroform, and a lipid film was formed in a rotavapor under vacuum at 40 °C. Dox was released from this composite in two steps: (1) passive diffusion of entrapped Dox and a small portion of Dox liposomes, and (2) an external thermal activation was used on Dox-loaded liposomes, which were irreversibly entrapped in the hydrogel (Figure 7). The effect of this controlled dosing system was in vitro tested on human ovarian carcinoma cells, and the results showed the potential ability of this system to reduce the exposure to sublethal doses of Dox while inhibiting the growth of cells with a short doubling time and avoiding the development of drug resistance.

Fat grafting is considered as a main regenerative medicine method; however, it often requires repeated procedures because of volume loss and high fat reabsorption. In 2024, Kadlecová et al. introduced an injectable thermo-sensitive drug delivery system by combining FGF2-STAB (a stable fibroblast growth factor 2 with a 21-day stability) with a thermo-sensitive FDA-approved hydrogel (itaconic acid-modified PLGA-PEG-PLGA copolymer), which showed higher stability (for 28 days) than wild-type FGF2 stability (just a few hours) [107]. In detail, the FGF2-STAB was encapsulated in biocompatible liposomes (with diameters of 85.73 ± 3.85 nm) prepared via the eco-friendly Mozafari method to guarantee pH protection. The liposomes made from DPPC (1,2-dipalmitoyl-sn-glycerol-3-phosphocholine, 2 wt %), and glycerol (3% *v*/*v*) with 68.6 ± 2.2% encapsulation efficiency allowed the controlled release of FGF2-STAB from the hydrogel. Rheological studies showed that the proteins and liposome-encapsulated proteins did not impact the mechanical stability of the hydrogel. The liposomes were an effective protective system for the delivery of FGF2-STAB. Also, these liposomes demonstrated to significantly enhance the release mechanism of FGF2-STAB, underscoring their potential in advanced therapeutic approaches. However, this study showed that the −COOH groups of the hydrogel were affected by the positive charge of the protein, which lowers the hydrolytic stability of the system; therefore, the carboxylic groups of the hydrogel should be replaced or masked to reduce the network–protein interactions for future applications.

*Chlamydia trachomatis* is considered as the most common cause of bacterial sexually transmitted infections among women, with more than 127 million new infections reported globally each year [108], resulting in serious reproductive tract complications, significantly affecting overall health and wellbeing. The limitations of currently used oral antibiotics and antimicrobial resistance issues require alternative and advanced therapeutic methods. In 2020, Jøraholmen proposed a novel liposome–CS hydrogel to deliver the natural polyphenol resveratrol (RES) for the localized treatment of *C. trachomatis* infections [109]. To synthesize the liposomes, RES (10 mg) was dissolved in ethanol and phosphatidylcholine (200 mg) in methanol. The solutions were mixed, and solvents were removed by evaporation. Both free RES and liposome–RES hydrogel prevented *C. trachomatis* propagation in a dose-dependent manner, studied by the common in vitro model using McCoy cells. However, for lower concentrations, liposome–RES hydrogel showed an enhanced anti-chlamydial effect with 78% and 94% inhibition for 1.5 and 3 µg/mL RES, respectively, compared to free RES (43% for 1.5 µg/mL; 72% for and 3 µg/mL). In addition, liposome–RES hydrogel exhibited an enhanced anti-inflammatory effect in a concentration-dependent inhibition of nitric oxide generation in LPS-induced macrophages. The use of such a delivery system provided enhanced antibacterial activity at lower concentrations (compared to the free drug) as well as applicability for vaginal administration, which could be a promising option for the localized treatment of *C. trachomatis* infections.

Other publications on liposome–biopolymer hydrogels for drug delivery applications are summarized in Table 4.

### 2.3. Liposomes Encapsulated in Synthetic Polymeric Hydrogels

In 2013, Vanić et al. prepared deformable propylene glycol-containing liposomes (DPGLs) encapsulating clotrimazole or metronidazole as efficient drug delivery vehicles to improve the treatment of vaginal microbial infections [154]. To obtain appropriate viscosities for vaginal administration, liposomes were entrapped into carbopol hydrogels. DPGLs diffused through the hydrogel matrix faster than conventional liposomes. Their in vitro drug release profiles were studied in conditions simulating human treatment and a sustained- and diffusion-based release was observed. The textural and rheological properties of DPGL hydrogels showed that the presence of DPGLs alone had no significant effect on the mechanical properties of the composite hydrogels. These results suggest the potential ability of DPGL hydrogels for the sustained release of antimicrobial drugs in the vagina.

Regarding vaginal drug delivery applications, Wei-Ze et al. developed a novel delivery system of post-expansile hydrogel foam aerosol of propylene glycol-containing liposomes (PG–liposomes) (PEHFL) [155]. A model drug, matrine (MT), was used to investigate the vaginal mucous membrane permeation properties of MT from PEHFL versus hydrogel foam aerosol (HFA), PG–liposome foam aerosol (PLFA) and hydrogel (HYG). Results showed the following: (i) 80.8 ± 2.6% of MT entrapment capacity in PG–liposomes; (ii) the PEHFL composite had a lagging swelling process after being spurted from a sealed container, and its swelling degree increased with the temperature of the surrounding environment, favoring a uniform drug diffusion in the vaginal canal, which tightly contacts vaginal walls; (iii) the mucoadhesive force of PEHFL foams was found to be 1460 ± 123 mN/cm^2^, which could remain 85 ± 11 min in vitro; (iv) the overall mean permeated MT through unit mass of porcine vaginal tissue from PEHFL was estimated as 7.59, 2.64 and 2.34 times higher than that from HYG, PLFA and HFA, respectively; and (v) the quantity of MT remaining in the vaginal tissue after 12 h was also significantly higher for PEHFL than for HYG, PLFA and HFA. These results demonstrated some advantages of PEHFL over conventional dosage forms, including enhancing the vaginal mucosa permeability of MT, uniform spreading in the vaginal canal, prolonged residence time at the site of administration and induction of MT delayed release. All these advantages suggest PEHFL as a promising delivery system for vaginal medications.

Pancreatic cancer is one of the most deadly malignancies with an all-stage 5-year survival rate of less than 5%, highlighting the urgent need for developing advanced and effective therapeutic methods [156]. Intratumoral delivery of anticancer drugs can divert the undesired drug distribution into non-target organs, consequently decreasing the systemic toxicity and increasing therapeutic efficacy. Thermo-sensitive injectable hydrogels have attracted interest because of their non-invasiveness over other localized implantable systems, with the ability to carry different drugs for site-specific delivery, prolonged drug action, reduced adverse side effects and improved patient compliance [157,158]. Mao et al. examined the delivery of paclitaxel (PTX), using a soybean phospholipid/cholesterol liposome and a P188-added P407 thermo-sensitive hydrogel (PTX–liposome hydrogel) as a local chemotherapy system against pancreatic cancer in a tumor-bearing mice model [159]. FDA-approved polymers, Poloxamer 407 (P407) and poloxamer 188 (P188) are most widely studied as temperature-sensitive polymers [160]. The prepared hydrogel composite had an appropriate sol-to-gel transition temperature. Studies on the morphology and particle size of the liposomes demonstrated this new dosage form allowed the physical stability of the drug without particle size growth or liposome precipitation. The in vitro release studies of PTX–liposome hydrogels showed a much slower release, compared to PTX–liposomes, with a good retention time inside the tumor tissue. The in vivo tests demonstrated a better balance between systemic safety and enhanced antitumor efficacy in PTX–liposome hydrogels, compared to other groups at equal drug dose. Therefore, this drug delivery system can provide a high local PTX concentration, sustained release, extended drug retention time inside of the tumor and, consequently, low toxicity towards healthy tissues.

In another study, Mourtas et al. studied the release of calcein (a fluorescent dye) and griseofulvin (GRF, a poorly water-soluble antifungal) from liposome hydrogels. Liposomes composed of cholesterol (DSPC/Chol) and phosphatidylcholine (PC) or distearoyl-glycero-PC, encapsulating GRF or calcein, were prepared by the thin-film hydration method [161]. After cleaning the drug-loaded liposomes, these were dispersed in different hydrogels (ehydroxylethyl-cellulose (HEC), carbopol 974 or a mixture of the two). GRF or calcein release was monitored by spectrophotometric and fluorescence techniques, respectively. The results showed that calcein release from liposome hydrogels is slower, compared to control gels, and can be further controlled and delayed by using rigid-membrane liposomes. Furthermore, calcein release was not influenced by the lipid amount (in the range from 2 to 8 mg/mL); therefore, solute loading can be controlled according to needs. Conversely, GRF release was affected by drug loading: at high loading levels, GRF was released with a constant rate from liposome hydrogels irrespective of liposome type (DSPC/Chol or PC). GRF and calcein release from control carbopol gels was faster, compared to HEC and mixture hydrogels, and the same was observed for calcein in liposome hydrogels. Rheological properties of carbopol hydrogels were found to be significantly different (compared to the other hydrogels), implying that these characteristics are important for drug diffusion from hydrogels.

Meloxicam (MX) is an effective hydrophobic non-steroidal anti-inflammatory drug clinically used to reduce pain and inflammation; however, its oral use can cause many adverse gastrointestinal issues. In 2020, Zhang et al. used a poloxamer P407-based hydrogel incorporated with transferosomes or flavosomes as a potential therapeutic vehicle for MX topical delivery [162]. In detail, MX was encapsulated in conventional liposomes, flavosomes and transferosomes made of phosphatidylcholine, cholesterol, cetylpyridinium chloride and flavonoids. Flavosomes are deformable liposomes containing flavonoids, specifically quercetin and dihydroquercetin. The different drug-loaded liposome formulations were incorporated into a poloxamer P407 hydrogel, due to its thermo-reversible gelation, solubilizing capacity, drug release characteristics and low toxicity. The developed deformable liposomes showed higher entrapment efficiency (as compared to conventional liposomes) with homogeneous vesicle sizes (less than 120 nm). They demonstrated improved permeability, compared to a liposome-free gel and a conventional liposome hydrogel, so they can be a promising alternative to MX conventional oral delivery. Interestingly, flavosome–hydrogel formulations showed the highest permeability into the deeper skin layers and decreased lag time, suggesting a potential faster on-site pain relief and anti-inflammatory effect.

**Figure 8:**
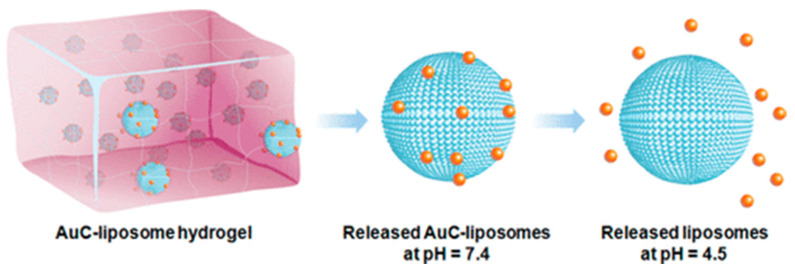
Schematic illustration of acrylamide hydrogels containing nanoparticle-stabilized liposomes for topical antimicrobial delivery. Carboxyl-modified gold nanoparticles (AuC) were adsorbed onto the outer surfaces of cationic liposomes to stabilize them against fusion. At physiological pH (pH = 7.4), AuC–liposomes are released from the hydrogel. When the pH drops below the pK_a_ value of the carboxylic group (pK_a_ ~ 5), AuC detach from the liposomes, resulting in the formation of bare liposomes with resumed fusion activity. Reprinted from ref. [168]; Copyright © 2014 American Chemical Society.

Liposomes have been introduced as a class of antimicrobial delivery vehicles thanks to their unique high drug loading capacity, biocompatible lipid materials, bilayer structure capable of fusing with microbial membranes and ready formulation properties [163,164,165]. However, the applications of small liposomes (below 100 nm) are often limited by their low stability due to spontaneous fusion, which results in drug loss or undesired release [166,167]. To stabilize liposomes against fusion prior to reaching their target, attaching small charged nanoparticles onto the liposome surfaces has become an effective approach. Therefore, nanoparticle-stabilized liposomes are considered effective drug delivery systems for the treatment of various infections. To evaluate the potential of this platform for clinical tests, Gao et al. combined carboxyl-modified gold nanoparticle-stabilized cationic liposomes made of EggPC (a zwitterionic phospholipid) and DOTAP (a cationic phospholipid) with acrylamide-based hydrogels (AuC–liposome hydrogel) to design a more effective drug delivery vehicle (Figure 8) [168]. The use of the hydrogel not only guarantees the structural stability of the nanoparticle-stabilized liposomes, but also provides controllable viscoelasticity and modulates liposome release rate. In this study, *Staphylococcus aureus* bacteria were used as model pathogens to demonstrate that nanoparticle-stabilized liposomes can be effectively released from the hydrogel matrix to the bacterial culture and that they subsequently combine with the bacterial membrane in a pH-dependent manner. The in vivo tests on mouse skin showed no observable skin toxicity within a 7-day treatment; therefore, this system holds great promise for topical applications against various microbial infections.

**Figure 9:**
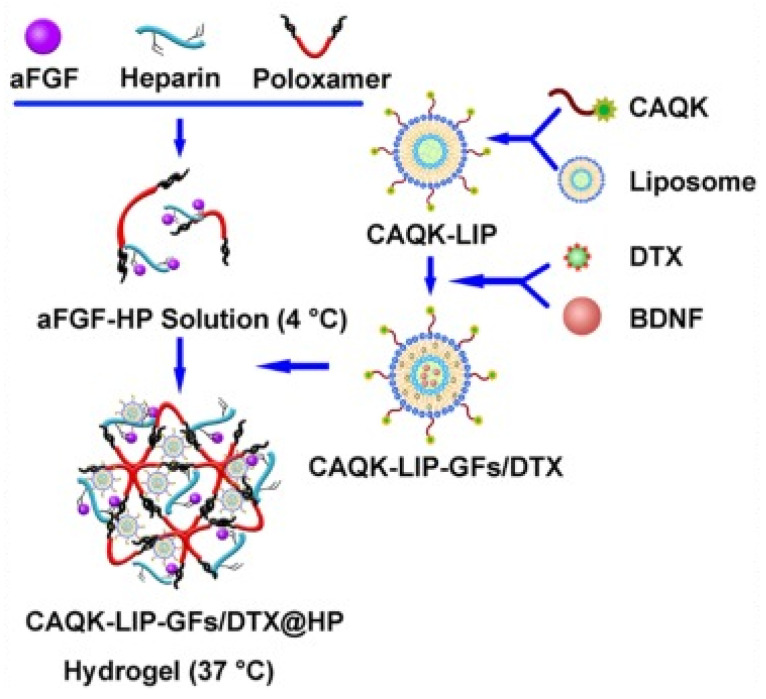
Schematic diagram of the CAQK-LIP-GFs/DTX-HP composite for targeted multiple-drug delivery system (aFGF: acidic fibroblast growth factor; CAQK: cysteine–alanine–glutamine–lysine; DTX: docetaxel; BDNF: brain-derived neurotrophic factor). Reprinted from ref. [179]; Creative Commons Attribution license.

Spinal cord injury (SCI) is one of the most harmful issues in medicine, and it is pathophysiologically characterized by a series of injurious biochemical cascades beyond the initial injury [169,170,171,172]. Advanced molecular and cellular therapies have been developed as promising approaches for targeting the secondary injury cascade of SCI [173,174,175]; however, they have not shown satisfactory therapeutic efficacy in clinical trials [176,177,178]. In SCI, it is vital to effectively deliver drugs, targeting multiple pathophysiological pathways. To this aim, Wang et al. developed a clinically reliable targeted delivery of multiple drugs to the SCI site and studied the mechanisms of neural recovery as well as the synergistic effect related to this combination therapy [179]. In this study, phospholipids and cholesterol-based liposomes were first modified with a scar-targeted tetrapeptide (cysteine–alanine–glutamine–lysine, CAQK), then used to encapsulate both an FDA-approved drug, docetaxel (DTX), and a brain-derived neurotrophic factor inside liposomes. Then, the drug-loaded liposomes were incorporated into a thermo-sensitive heparin-modified poloxamer injectable hydrogel (HP) with affinity-bound acidic fibroblast growth factor (aFGF–HP) for local administration to the SCI target site in a rat model (Figure 9). The specificity of the CAQK-LIP-GFs/DTX-HP composite towards the injured site was studied using fluorescence imaging, along with multiple evaluations, including magnetic resonance imaging and biotin dextran amine anterograde tracing to detect the synergistic effects and the related mechanisms of CAQK- LIP-GFs/DTX-HP both in vitro and in vivo. The results showed the effective delivery of multiple drugs to the injured site, supporting neuro-regeneration by improving neuronal survival and plasticity, which affords a more permissive extracellular matrix environment with enhanced regeneration potential. Also, this combination therapy promoted mitochondrial transport along the regenerating axon and axonal regeneration via moderation of microtubule function. This novel targeted multi-drug delivery system possesses acceptable cytocompatibility, biocompatibility and thermo-sensitivity, which offer promising translational prospects for clinical SCI treatment.

**Figure 10:**
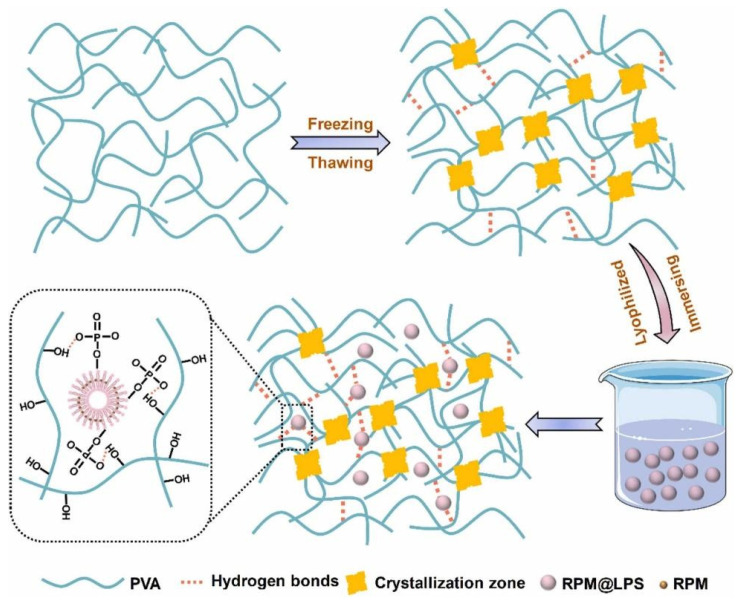
Schematic illustration of rapamycin-loaded hydrophilic hydrogel coating to obtain RPM–liposome/PVA-c-PP. Reprinted from ref. [177]; Copyright 2023, Elsevier.

Hernia is a common surgical issue that refers to tissues or visceral organs that protrude through a weakness or damaged wall area [180,181]. Polypropylene (PP) mesh has been frequently used in hernia repair as a prosthetic material due to its excellent mechanical properties and biocompatibility. However, abdominal adhesion between the PP mesh and visceral tissues is still a major issue; therefore, Wei et al. designed an anti-adhesive PP mesh using poly(vinyl alcohol) (PVA)–hydrogel and a liposome-based drug delivery system. First, a PVA–hydrogel coating was performed on the surface of PP mesh using freezing–thawing processing cycles to form PVA-c-PP. Then, the coated PP mesh was immersed in a rapamycin (RPM)-loaded liposome (made from soybean lecithin and cholesterol) solution to obtain the final anti-adhesion mesh: RPM–liposome/PVA-c-PP (Figure 10) [177]. RPM was used because of its various functions, including strong anti-angiogenesis, immunosuppression, anti-fibrosis and low side effects [182]. RPM has been commonly used in the treatment of lung transplantation, kidney transplantation and autoimmune diseases. It was shown that the hydrogel coating can remain on the PP surface even after being immersed in PBS solution at 40 °C for up to one month. In vitro cell tests demonstrated the excellent cytocompatibility of this composite and its potential to inhibit cell adhesion. Moreover, in vivo experiments confirmed the enhanced anti-adhesive effects of RPM–liposome/PVA-c-PP mesh, compared to the uncoated PP mesh throughout the duration of implantation. In addition, the results proved that the modified mesh has lower inflammation responses and significantly looser fibrous tissue surrounding the PP filaments, compared to pristine PP.

## 3. Conclusions: Current Challenges and Future Trends

Hydrogels have found numerous uses for drug delivery applications thanks to their inherent properties of biodegradability and biocompatibility, and many therapeutic molecules have been successfully entrapped into different hydrogel carriers. These soft materials have substantial potential to be employed for various pharmaceutical applications; however, there are still many challenges and hurdles that need to be surpassed before clinically approving a hydrogel product. For instance, one of the major concerns is uncontrolled drug release from the polymer matrix, which may induce undesirable side effects. Nevertheless, in recent years, the FDA has approved several commercialized hydrogel-based products such as Revanesse^®^ VersaTM, Belotero balance^®^, Teosyal^®^ RHA, SpaceOAR^®^, TraceIT^®^ and Radiesse^®^ [183,184]. A successful future is ahead for marketed hydrogel products, as the needs for patient-specific healing treatment continue to grow day by day. It should be mentioned that hydrogels developed for controlled drug delivery often present several limitations, and to address these issues and improve their efficacy, the integration of hydrogels and liposomes could represent a promising strategy to minimize fast drug release. Liposomes have been the most successful family within the field of nanomedicine, and a number of liposomal–drug formulations have reached the market. In liposome–hydrogel formulations, both hydrogels and liposomes can improve each other structurally, for instance, the hydrogel can modify mechanical stability and membrane integrity of the encapsulated liposomes. In this particular approach, a number of parameters should be optimized such as particle size, lipid composition of liposomes and morphology and surface charge of both hydrogels and liposomes to efficiently encapsulate hydrophobic and/or hydrophilic therapeutic agents. Remarkably, the protecting effect of liposomes to drugs can be further improved through their incorporation with hydrogels. More importantly, the possibility of tuning either liposome and/or hydrogel structures to be sensitive to environmental stimuli (e.g., pH, light, temperature) makes them promising candidates for development of novel drug delivery systems. Three types of hydrogels containing liposomes discussed in this review have proven their efficacy in controlled drug delivery. In conclusion, targeted controlled drug delivery systems are of great significance to achieve a huge breakthrough in treating many diseases, specifically for cancer therapy. Advanced liposomes–hydrogels can target tumor sites, releasing smart nanovesicles (like liposomes) and encapsulating anticancer drugs in a sustained manner, which leads to a highly localized drug concentration in the tumor environment, while preserving healthy cells and consequently minimizing the side effects of the therapy.

As a perspective view, more studies should be conducted to study the possibility of transferring liposome–hydrogel composites from the lab-scale to industrial applications by taking into consideration several parameters such as liposomes’ potential leakage, stability issues, cytotoxic effects, effective sterilization methods, batch-to-batch reproducibility and scale-up. Despite undeniable progress and benefits of these hybrid materials shown both in in vitro and in vivo experiments, no liposome–hydrogel-based material has been launched into clinical trials so far. This may be mainly due to expensive manufacturing processes, stability issues and the need for these materials to be implanted in most cases because of their dimensions and elastic properties. These disadvantages might still limit the clinical use and large-scale preparation of such liposome–hydrogel materials in the future.

## Figures and Tables

**Table 1:** List of liposomal formulations approved for clinical use by FDA and EMA, excluding lipid–drug complexes [10].

Application	Product Name	API	Approved Year/Area	Therapeutic Indications
Cancer therapy	Doxil^®^/CaelyxTM	Doxorubicin hydrochloride (DOXHCl)	1995 (US) 1996 (EU)	Breast and ovarian cancer, Kaposi’s sarcoma
	DaunoXome^®^	Daunorubicin	1996 (US, EU)	Kaposi’s sarcoma
	Onivyde^®^	Irinotecan hydrochloride trihydrate	1996 (US) 2016 (EU)	Pancreatic adenocarcinoma
	Myocet^®^	Doxorubicin	2000 (EU)	Breast cancer
	Mepact^®^	Mifamurtide	2009 (EU)	Osteosarcoma
	Marqibo^®^	Vineristine	2012 (US)	Leukemia
	Vyxeos^®^	Daunorubicin + cytrabine	2017 (US) 2018 (EU)	Leukemia
	Zolsketil^®^	Doxorubicin	2022 (EU)	Breast and ovarian cancer, multiple myeloma, Kaposi’s sarcoma
Other applications	AmBisome^®^	Amphotericin B	1997 (US, EU)	Fungal infections
	DepoCyt^®^	Cytarabine	1999 (US) 2001 (EU)	Lymphomatous meningitis
	Visudyne^®^	Verteporphin	2000 (US, EU)	Age-related macular degeneration
	DepoDur^®^	Morphine sulfate	2004 (US, EU)	Pain management
	Arikayce^®^	Amikacin	2018 (US, EU)	Lung infections
	Exparel^®^	Bupivacaine	2020 (EU)	Anesthesia
Vaccines	Epaxal^®^	Inactivated hepatitis A virus (RG-SB strain)	1994 (EU)	Hepatitis A
	Inflexal V^®^	Influenza virus surface antigens (haemagglutinin and neuraminidase), Virosomal, 3 different strains	1997 (EU)	Influenza
	MosquirixTM	Proteins found on the surface of the Plasmodium falciparum parasites and the hepatitis B virus	2015 (EU)	Malaria
	Shingrix^®^	Recombinant varicella-zoster virus glycoprotein E	2017 (US) 2018 (EU)	Shingles and post-herpetic neuralgia
	COMIRNATY™	mRNA	2021 (US, EU)	COVID-19
	SPIKEVAX™	mRNA	2022 (US, EU)	COVID-19

**Table 3:** Main advantages and disadvantages of liposomes [35].

Advantages	Disadvantages
Increased efficiency and therapeutic index of drugs	Low solubility
Enhanced drug stablility	Short half-life
Non-toxic, flexible, biocompatible, biodegradable and non-immunogenic	Possible phospholipid oxidation and hydrolysis-like reactions
Decreased toxicity to the encapsulated drug	Leakage and fusion of encapsulated drugs
Reduction in the exposure of sensitive tissues to toxic drugs	High production costs
Site avoidance effect	Low stability
Improved pharmacokinetics	

**Table 4:** Publications on liposome–biopolymer hydrogels used for drug delivery applications.

Biopolymer	Liposomes	Delivered Agent	References
Chitosan (CS)	phosphatidylcholine liposomes of various sizes	Mupirocin	[110,111,112,113,114,115,116]
Gelatin	vesicles made of sodium oleate	Calcein	[117,118,119,120,121,122,123,124,125]
Dextran	SOPC/DOTAP liposomes	N/A	[126,127,128]
Hyaluronic acid	thermo-responsive liposomes (i.e., dipalmitoylphosphatidylcholine (DPPC) and dimyristoylphosphatidyl choline (DMPC))	horseradish peroxidase	[117,129,130,131,132,133,134,135]
Alginate	dipalmitoylphosphatidylcholine liposomes	cytochrome-c	[136,137,138,139,140,141,142,143,144,145,146,147,148]
Carrageenan	niosomes based on a non-ionic surfactant molecule and cholesterol	meloxicam	[132,149,150]
Methylcellulose	niosomes based on two non-ionic surfactants (span 20 and span 60) and cholesterol	acyclovir	[151]
Xanthan gum	non-ionic surfactant niosomes based on Tween 20 and cholesterol	caffeine, ibuprofen	[152,153]

## Data Availability

Not applicable.
